# Effectiveness and safety of single transseptal ablation for atrial fibrillation in real‐word practice

**DOI:** 10.1002/clc.23574

**Published:** 2021-02-22

**Authors:** Yoon Jung Park, Hee Tae Yu, Tae‐Hoon Kim, Jae‐Sun Uhm, Boyoung Joung, Moon‐Hyoung Lee, Hui‐Nam Pak

**Affiliations:** ^1^ Department of Cardiology Yonsei University Health System Seoul Republic of Korea

**Keywords:** atrial fibrillation, catheter ablation, complication, single groin, single transseptal puncture

## Abstract

**Background:**

We have previously reported that unilateral groin‐single transseptal (ST) ablation in patients with paroxysmal atrial fibrillation (AF) was safe and significantly reduced patient discomfort compared with bilateral groin‐double transseptal (DT) ablation.

**Hypothesis:**

In the present study, we hypothesized that ST ablation would be as effective and safe as DT ablation in real‐world practice like previous study. Among the 1765 consecutive patients in the Yonsei AF ablation cohort from October 2015 to January 2020, 1144 patients who underwent radiofrequency ablation were included for the analysis. Among them, 450 underwent ST ablation and 694 underwent DT ablation.

**Results:**

The total procedure time, ablation time, and fluoroscopy time were longer in the ST group than in the DT group (*p* < .05 for all). The hospital stay after catheter ablation was 1.3 ± 1.1 days which was longer in DT group than ST group (*p* = .001). No significant difference was observed in the complication rate (*p* = .263) and AF‐free survival rate (log‐rank *p* = .19) between the groups. However, after excluding patients who used antiarrhythmic drugs when AF recurred, the AF‐free survival rates were lower in the DT group than in the ST group before and after propensity score matching (log‐rank *p* = .026 and .047, respectively).

**Conclusion:**

Although the ST approach increases the procedure time compared with the DT approach owing to the need for more frequent catheter exchanges, the ST approach is a feasible and safe strategy for AF ablation in terms of rhythm outcomes and risk of complications.

## 1 INTRODUCTION

Catheter ablation is effective in rhythm control of atrial fibrillation (AF) and maintaining sinus rhythm.^1^, ^2^, ^3^, ^4^, ^5^ After a study identified triggers of paroxysmal AF in the pulmonary veins (PVs), catheter ablation of AF has become a more common treatment method.^6^, ^7^ Complete PV isolation is a well‐proven endpoint for catheter ablation by encircling the PVs.^8^, ^9^ To approach the PVs via the left atrium (LA) during AF ablation, transseptal puncture is performed.^10^ Although some complications have been reported, such as cardiac tamponade, stroke, and persistence of the septal defect after transseptal puncture,^11^, ^12^, ^13^ several reports have shown the safety of transseptal puncture.^14^, ^15^ During PV isolation, most operators use the double transseptal (DT) approach for simultaneous PV potential monitoring and ablation.

We have previously compared unilateral groin (UG) puncture‐single transseptal (ST) ablation with the conventional bilateral groin (BG) puncture‐double transseptal (DT) ablation in a prospective randomized trial.^16^ No differences in clinical outcome and complications were found between the two groups. However, patient's discomfort about hemostasis was reduced in the UG‐ST group than the BG‐DT group. The number of patients in our previous prospective study was relatively small. Therefore, in the present study, we sought to assess the effectiveness and safety of ST ablation compared to those of DT ablation in patients with AF in real‐world clinical practice.

## 2 METHODS

### 2.1 Study population

**FIGURE 1:**
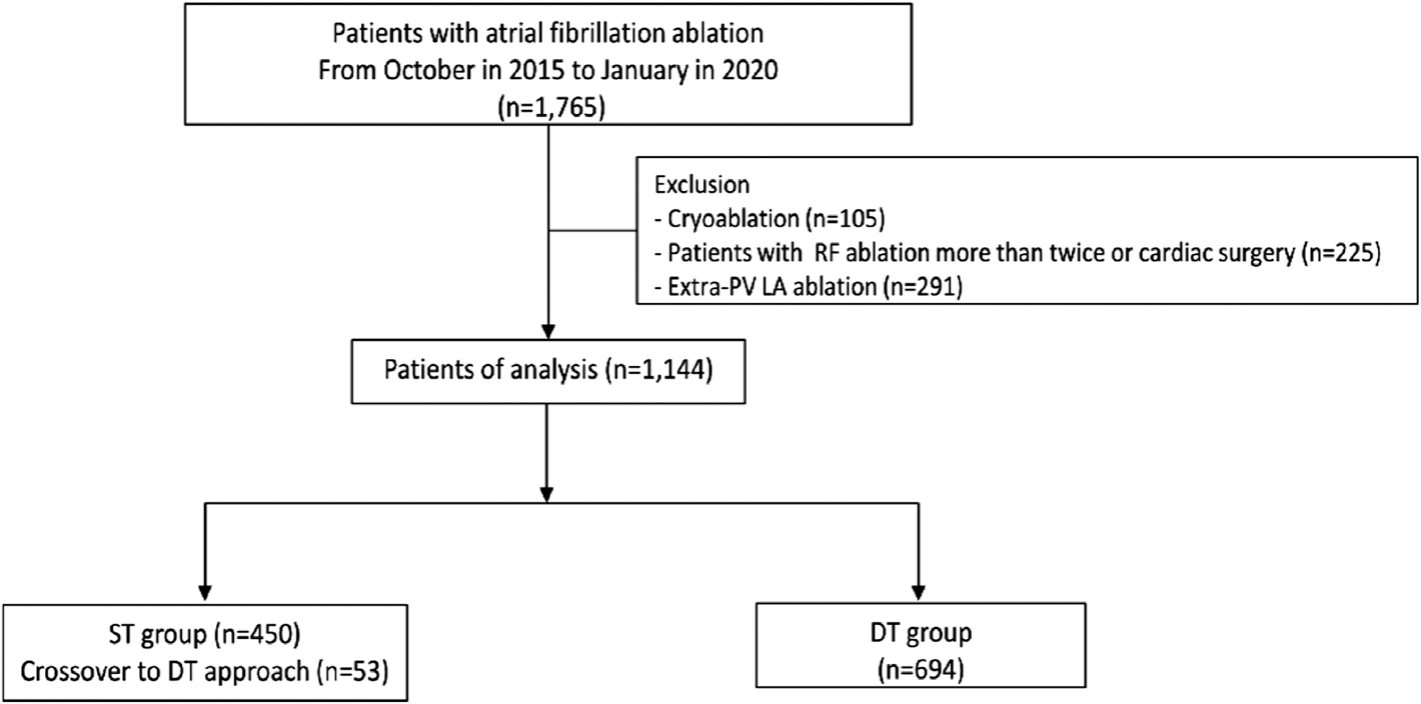
Flow diagram of the study population. DT, double trans‐septal; LA, left atrial; PV, pulmonary vein; ST, single trans‐septal

The study protocol adhered to the Declaration of Helsinki and was approved by the Institutional Review Board of Yonsei University Health System. All patients provided written informed consent for inclusion in the Yonsei AF Ablation Cohort Database (registered at clinicaltrials.gov as NCT02138695). Among the 1765 consecutive patients in the Yonsei AF ablation cohort from October 2015 to January 2020, those who underwent de novo radiofrequency catheter ablation for circumferential PV isolation (CPVI) were included in the current analysis (Figure 1). The exclusion criteria were as follows: (1) valvular AF, (2) structural heart disease other than left ventricular hypertrophy, (3) cryoablation, (4) history of a previous AF ablation or cardiac surgery, and (5) extra ablation other than PV ablation in the LA. The remaining 1144 patients (73.8% male, mean age 58.3 ± 10.6 years, 75% with paroxysmal AF) were divided into the ST and DT groups. Choice of ST or DT approach was dependent on operator preference. If PV isolation could not be achieved in any patient in the ST group after three‐times of touch‐up ablations, the patient was crossed over to the DT group.

### 2.2 Electrophysiological mapping and ablation

In the DT group, both the groins were used. A 14‐Fr venous sheath (Fast‐Cath™ Trio, St. Jude Medical Inc.) was introduced into the left femoral vein, and two long transseptal long sheaths (Schwartz left‐1, 8‐Fr; Abbott Inc.) were introduced into the right femoral vein. In the ST group, a single groin was used. A 14‐Fr venous sheath and a transseptal long sheath were introduced into the right femoral vein. Ablation was performed in all patients using three‐dimensional (3D) electroanatomical mapping (NavX; St Jude Medical Inc., Minnetonka, MN, USA) merged with 3D spiral computed tomography (CT). Systemic anticoagulation was performed with intravenous heparin to maintain an activated clotting time of 350–400 s during the procedure.

For PV ablation, continuous circumferential lesions were created at the level of the LA antrum, encircling the right and left PVs under the guidance of the NavX system using an open‐irrigated, 3.5 mm‐tip deflectable catheter at 30–35 W and 45°C (Coolflex [St. Jude Medical Inc.]). CPVI was performed in all patients. The endpoints of CPVI and cavotricuspid isthmus (CTI) block were defined on the basis of bidirectional pacing. The procedure ended when no immediate recurrence of AF was observed within 10 min after cardioversion with an isoproterenol infusion (5–10 μg/min). Non‐PV foci under an isoproterenol infusion were also ablated.^17^

### 2.3 Postablation follow‐up

Patients without antiarrhythmic medications were discharged after the procedure unless early recurrence of AF/atrial tachycardia (AT) or symptomatic frequent atrial premature beats were evident. The patients regularly visited the outpatient clinic at 1, 3, 6, and 12 months after catheter ablation and every 6 months thereafter or whenever symptoms occurred. All patients underwent electrocardiography (ECG) at each visit and 24 h Holter recordings at 3 and 6 months after catheter ablation and every 6 months thereafter according to the guideline.^18^ Holter monitoring or event monitor recordings were obtained when the patients reported symptoms of palpitation suggestive of arrhythmia recurrence. AF recurrence was defined as any episode of AF or AT lasting for at least 30 s. Any ECG documentation of an AF recurrence within a 3‐month blanking period was diagnosed as an early recurrence, and an AF recurrence >3 months after the procedure was diagnosed as a clinical recurrence.

### 2.4 Statistical analysis

For the comparison of baseline variables, continuous variables were analyzed using Student's *t*test or analysis of variance, as appropriate. Categorical variables were analyzed using Pearson's chi‐square test. The AF recurrence‐free survival over time was compared between the ST and DT groups using Kaplan–Meier analysis. Propensity score matching was performed to adjust for some variables in the two groups. Statistical analysis was performed using SPSS for Windows (version 25; IBM Corp., Armonk, NY, USA) and R statistical software. A *p*‐value of <.05 was considered statistically significant.

## 3 RESULTS

### 3.1 Baseline and procedure‐related characteristics

Among 1144 patients undergoing AF ablation with PV isolation, 450 patients underwent ST ablation and 694 patients underwent DT ablation. A total of 844 (73.8%) patients were male and the mean age of the patients was 58.3 ± 10.6 years (Table 1). The mean age was higher and the proportion of men was lower in the ST group than in the DT group. No significant differences were observed in comorbidities and echocardiographic parameters between the groups. The total procedure time, ablation time, and fluoroscopy time were longer in the ST group than in the DT group (Table 2). The mean hospital stays after radiofrequency catheter ablation (RFCA) was 1.3 ± 1.1 days which was longer in DT group than ST group (*p* = .001 before propensity score matching and *p* = .004 after propensity score matching).

**TABLE 1:** Baseline clinical characteristics

	Before propensity score matching				After propensity score matching			
	Overall (*n* = 1144)	ST group (*n* = 450)	DT group (*n* = 694)	*p*‐value	Overall (*n* = 826)	ST group (*n* = 413)	DT group (*n* = 413)	*p*‐value
Age (y)	58.3 ± 10.6	59.5 ± 11.1	57.6 ± 10.2	.003	59.5 ± 10.7	59.6 ± 11.0	59.3 ± 10.4	.623
Male sex	844 (73.8)	316 (70.2)	528 (76.1)	.028	587 (71.1)	294 (71.2)	293 (70.9)	.939
BMI (kg/m^2^)	25.3 ± 3.4	25.0 ± 3.4	25.5 ± 3.4	.041	25.2 ± 6.0	25.0 ± 3.4	25.5 ± 7.7	.294
Paroxysmal AF	849 (75.0)	336 (76.7)	513 (73.9)	.291	635 (76.9)	318 (77.0)	317 (76.8)	.934
Comorbidities								
Heart failure	114 (10.0)	43 (9.6)	71 (10.2)	.710	76 (9.2)	35 (8.5)	41 (9.9)	.470
Hypertension	518 (45.3)	196 (37.8)	322 (46.4)	.345	360 (43.6)	179 (43.3)	181 (43.8)	.888
Diabetes mellitus	184 (16.1)	63 (14.0)	121 (17.4)	.122	125 (15.1)	59 (14.3)	66 (16.0)	.497
Stroke or TIA	110 (9.6)	42 (9.3)	68 (9.8)	.794	76 (9.2)	39 (9.4)	37 (9.0)	.810
Vascular disease	54 (4.7)	25 (5.6)	29 (4.2)	.283	47 (5.7)	23 (5.6)	24 (5.8)	.881
CHA_2_DS_2_‐VASc score	1.6 ± 1.4	1.7 ± 1.5	1.5 ± 1.3	.056	3.6 ± 1.5	3.6 ± 1.5	3.6 ± 1.4	.981
Echocardiography								
LA diameter (mm)	40.6 ± 6.0	40.5 ± 5.7	40.7 ± 6.2	.579	40.5 ± 6.0	40.4 ± 5.7	40.5 ± 6.2	.802
LAVI (ml/m^2^)	36.9 ± 12.5	36.8 ± 13.1	37.0 ± 12.1	.718	36.6 ± 11.5	36.2 ± 11.5	37.0 ± 11.6	.331
LVEF(%)	63.6 ± 8.1	64.1 ± 8.1	63.3 ± 8.2	.106	64.2 ± 8.1	64.3 ± 8.0	64.1 ± 8.3	.662
E/Em	9.8 ± 4.0	9.9 ± 3.6	9.8 ± 4.3	.632	9.9 ± 3.7	9.8 ± 3.6	9.9 ± 3.9	.773
LVEDD (mm)	49.8 ± 4.5	49.9 ± 4.6	49.8 ± 4.5	.785	49.6 ± 4.4	49.9 ± 4.6	49.3 ± 4.3	.076

*Note*: Variables are presented as mean ± SD or count (percentage).

Abbreviations: AF, atrial fibrillation; BMI, body mass index; DT, double transseptal; E/Em, early mitral inflow velocity to early diastolic mitral annular velocity ratio; LA, left atrial; LAVI, left atrial volume index; LVEF, left ventricular ejection fraction; LVEDD, left ventricular end diastolic dimension; ST, single transseptal; TIA, transient ischemic attack.

**TABLE 2:** Procedure‐related characteristics and outcomes

	Before propensity score matching				After propensity score matching			
	Overall (*n* = 1144)	ST group (*n* = 450)	DT group (*n* = 694)	*p*‐value	Overall (*n* = 826)	ST group (*n* = 413)	DT group (*n* = 413)	*p*‐value
Procedure time (min)	139.0 ± 50.0	156.7 ± 34.2	127.4 ± 55.0	<.001	145.2 ± 46.9	156.7 ± 33.4	133.7 ± 55.0	<.001
Ablation time (min)	56.5 ± 21.5	64.4 ± 19.3	51.5 ± 21.4	<.001	59.0 ± 21.0	64.5 ± 18.8	53.6 ± 21.8	<.001
Fluoroscopy time (min)	27.1 ± 11.7	28.2 ± 9.7	26.4 ± 12.8	.009	27.7 ± 11.4	28.1 ± 9.5	27.3 ± 13.1	.341
Hospital stay after RFCA (day)	1.3 ± 1.1	1.2 ± 0.8	1.4 ± 1.2	.001	1.3 ± 1.2	1.2 ± 0.8	1.4 ± 1.5	.004
Follow up (months)	17.8 ± 13.8	15.6 ± 12.9	19.1 ± 14.2.	<.001	18.2 ± 13.7	16.0 ± 12.9	20.3 ± 14.3.	<.001
Early recurrence	291 (25.4)	90 (20.0)	201 (29.0)	.001	199 (24.1)	86 (20.8)	113 (27.4)	.028
Clinical recurrence	221 (19.3)	72 (16.0)	152 (21.5)	.022	152 (18.4)	66 (16.0)	86 (20.8)	.073
AAD before AF recurrence	371 (35.5)	107 (26.2)	264 (41.4)	<.001	249 (32.6)	101 (26.7)	148 (38.3)	.001
AAD after AF recurrence	325 (31.1)	103 (25.2)	222 (34.9)	.001	221 (28.9)	98 (25.9)	123 (31.9)	.070
Complications	42 (3.7)	20 (4.4)	22 (3.2)	.263	32 (3.9)	18 (4.4)	14 (3.4)	.471
Cardiac tamponade/hemopericardium	17 (1.5)	6 (1.3)	11 (1.6)	.731	14 (1.7)	6 (1.5)	8 (1.9)	.590
AE fistula	1 (0.1)	0 (0)	1 (0.1)	.420	1 (0.1)	0 (0)	1 (0.1)	.317
Stroke	3 (0.3)	0 (0)	3 (0.4)	.163	2 (0.2)	0 (0)	2 (0.5)	.157
PV stenosis	3 (0.3)	3 (0.7)	0 (0)	.031	2 (0.2)	2 (0.5)	0 (0)	.157
SSS/AV block	6 (0.5)	4 (0.9)	2 (0.3)	.169	5 (0.6)	4 (1.0)	1 (0.2)	.178
Femoral arteriovenous fistula	5 (0.4)	3 (0.7)	2 (0.3)	.343	2 (0.2)	2 (0.5)	0 (0)	.157
Puncture site hematoma	3 (0.3)	1 (0.2)	2 (0.3)	.831	2 (0.2)	1 (0.2)	1 (0.2)	1.000
Others	4 (0.3)	3 (0.7)	1 (0.1)	.144	4 (0.5)	3 (0.7)	1 (0.2)	.316

*Note*: Variables are presented as mean ± standard deviation or count (percentage).

Abbreviations: AAD, antiarrhythmic drug; AE, atrioesophageal; AF, atrial fibrillation; AV, atrioventricular; DT, double transseptal; PV, pulmonary vein; RFCA, radiofrequency catheter ablation; SSS, sick sinus syndrome; ST, single transseptal.

The rate of procedure‐related complications was 3.7% (42 of 1144 patients). Most of the complications were not significantly different between the groups, including femoral puncture site‐related complications. PV stenosis occurred in three patients, and one of them was performed balloon angioplasty because of significant stenosis. Although PV stenosis was more common in the ST group than in the DT group, statistical significance could not be estimated because of the small incidence. Stroke occurred in three patients; all patients had undergone DT ablation. One of them was transient ischemic attack and all patients had no significant sequelae after stroke.

### 3.2 Clinical rhythm outcomes

**FIGURE 2:**
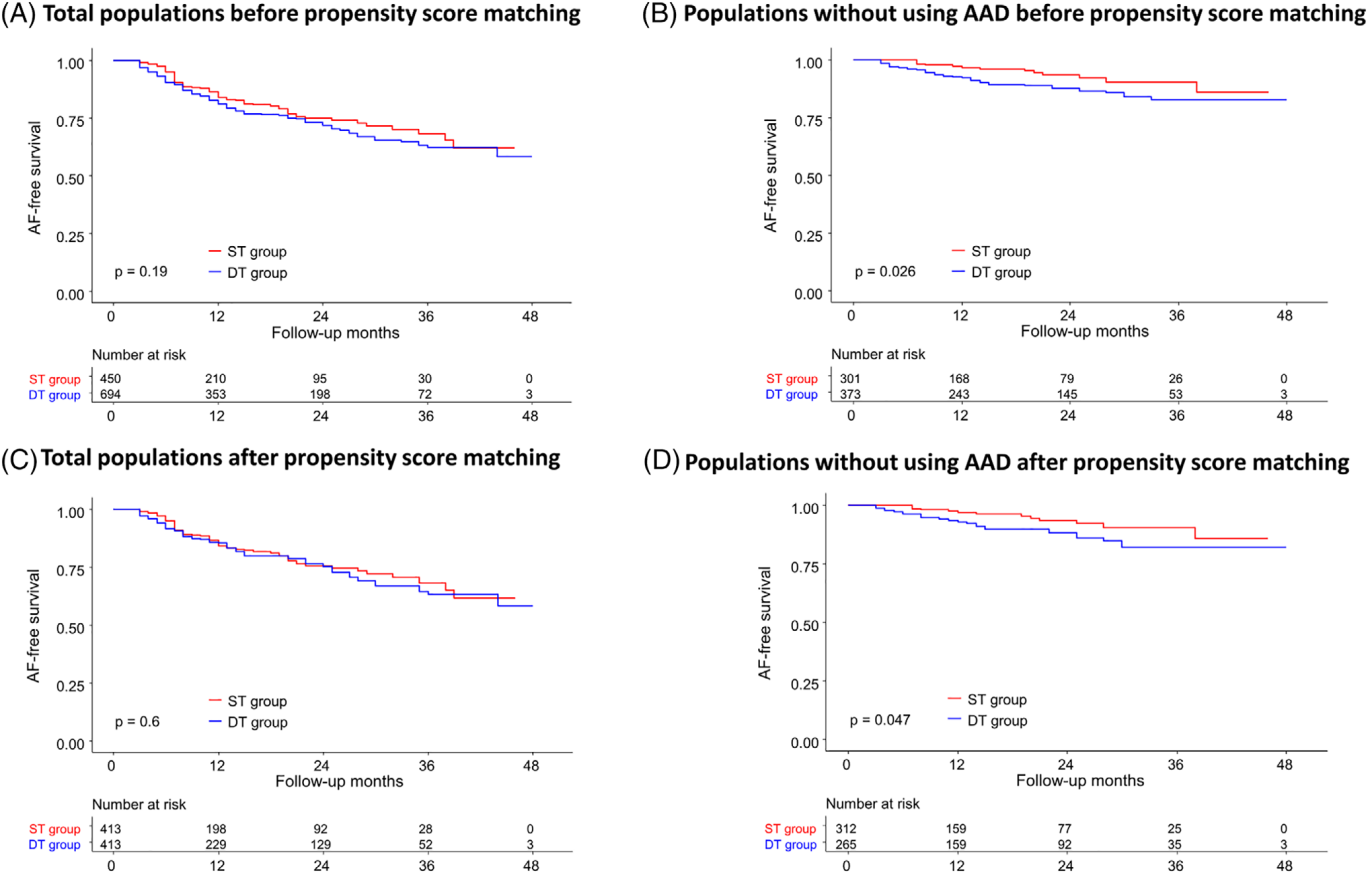
Kaplan–Meier curve for AF recurrence‐free survival before propensity score matching (A), AF recurrence‐free survival off AAD before propensity score matching (B), AF recurrence‐free survival after propensity score matching (C) and AF recurrence‐free survival off AAD after propensity score matching (D). ST, single trans‐septal; DT, double trans‐septal; AAD, anti‐arrhythmic drug

The mean follow‐up duration was 17.8 ± 13.8 months (Table 2). Clinical recurrence of AF was observed in 221 (19.3%) of 1144 patients. Among patients with AF recurrence, 371 (35.5%) used antiarrhythmic drugs (AADs) before recurrence. Patients in the DT group used AADs more frequently than those in the ST group before clinical recurrence. The AF recurrence‐free survival rate was not significantly different between the ST group and the DT group in the Kaplan–Meier analysis (log‐rank *p* = .190, Figure 2(A)). However, after excluding patients who used AADs when AF recurred, the AF recurrence free‐survival rate was lower in the DT group than in the ST group (log‐rank *p* = .026, Figure 2(B)).

Among patients who recurred AF, 77 patients performed redo‐ablation. (Table 3). A total of eight patients (10.4%) observed 4PV isolation when PV potential was checked in redo‐RFCA. There was no significant difference in PV reconnection between ST group and DT group.

**TABLE 3:** Redo‐ablation characteristics

	Overall (*n* = 77)	ST group (*n* = 28)	DT group (*n* = 49)	*p*‐value
PV reconnection, *n* (%)				.646
4PV isolation	8 (10.4)	4 (14.3)	4 (8.2)	
PV reconnection	69 (89.6)	24 (85.7)	45 (91.8)	
1 reconnection	14 (18.2)	5 (17.9)	9 (18.4)	
2 reconnection	19 (24.7)	6 (21.4)	13 (26.5)	
3 reconnection	14 (18.2)	5 (17.9)	9 (18.4)	
4 reconnection	22 (28.6)	8 (28.6)	14 (28.6)	

*Note*: Variables are presented as count (percentage).

Abbreviations: DT, double transseptal; PV, pulmonary vein; ST, single transseptal.

### 3.3 Outcomes after propensity score matching

After propensity score matching to balance the baseline characteristics between the two groups, the procedure time, ablation time, and fluoroscopy time were still significantly longer in the ST group than in the DT group (Table 2). No significant difference in the rate of complications (4.4% vs. 3.4%, *p* = .471) was found between the ST and DT groups. The Kaplan–Meier analysis of the AF‐free survival rate also showed no significant difference between the two groups after propensity score matching (log‐rank *p* = .600, Figure 2(C)). The AF‐free survival rate was also lower in the DT group than in the ST group after excluding patients with AAD use in propensity matching (log‐rank *p* = .047, Figure 2(D)).

## 4 DISCUSSION

### 4.1 Major findings

Our study divided patients undergoing AF ablation based on the approach used (ST and DT) to determine the feasibility and safety of the ST approach. Although the procedure time was increased in the ST group, the incidence of complications was not significantly different between the ST and DT groups. The AF‐free survival rates in the Kaplan–Meier curve were not significantly different between the two groups. After excluding patients who used AADs when AF recurred, the AF‐free survival rates without AAD use were lower in the DT group than in the ST group before and after propensity score matching.

### 4.2 Feasibility of the ST approach

ST or DT puncture was used for ablation of AF. Haissaguerre et al was initially introduced technique using ST puncture and double transseptal catheterization.^7^, ^19^ A previous retrospective study^15^ and other report^20^ have showed that ST puncture is safe and could be used as an alternative to reduce the complications of performing a second transseptal puncture, such as pericardial tamponade, coronary artery spasm by mechanical irritation,^21^ and air embolism.^22^ However, ST puncture and double transseptal catheterization sometimes causes the problem of catheter manipulation.^10^ Single transseptal catheterization used in our study resolved the problem of catheter manipulation. The rate of complications in our study was not significantly different between the two groups. Although we could not conclude that ST puncture is safer than DT puncture, we could propose that ST puncture is as safe as DT puncture.

No significant difference was observed between the ST and DT groups in terms of AF recurrence before and after propensity score matching. The use of AADs after ablation might affect the AF recurrence. After removing the effect of AADs by excluding patients who used AADs when AF recurred, the risk of AF recurrence was higher in the DT group than in the ST group. The operators might have been more careful and took more time to complete CPVI in the ST group because they could not see the remnant PV potentials spontaneously to confirm PV isolation, which could have affected the difference in the risk of AF recurrence between the two groups.

The procedure time and ablation time were higher in the ST group than in the DT group. The time to exchange the ablation catheter to a mapping catheter might have affected the procedure time extension in the ST group. ST group performed more CTI ablation than DT group (98.9% versus 65.9%). It could have impacted the procedure and fluoroscopy times. As the ST approach was used at our center from 2015, it took time for achieving the learning curve, whereas the DT approach has been a routine common approach in AF ablation.

### 4.3 Reduction of postprocedural discomfort after the ST approach

AF ablation is a complex procedure that requires multiple catheter positioning and a relatively long procedure time, and continuous intra‐ and peri‐procedural anticoagulation. The advantage of ST puncture is that it uses a single groin, which could reduce the time of hemostasis. A previous report compared the safety and feasibility of UG‐ST ablation with those of BG‐DT ablation in patients with paroxysmal AF.^16^ A survey regarding patient discomfort showed that UG‐ST reduced the discomfort with respect to postprocedural hemostasis compared with BG‐DT. When both groins are used, patients usually experience discomfort with back and leg pain during postprocedural hemostasis because of immobility for at least 6 h because of the possibility of bleeding. In patients in the ST group, a single groin was used during the procedure, allowing them to move the other leg immediately after the procedure, thus reducing the discomfort during hemostasis.

Hospital stay after RFCA was significantly reduced in ST group compared to DT group. Reducing patient's discomfort during hemostasis might be helpful to reduce hospital stay after RFCA.

### 4.4 Potential complications of the ST approach

In the ST approach, the risk of stroke might increase because catheters are exchanged several times during the procedure. Therefore, we always irrigated the sheath with saline before catheter exchange to remove clots and air. However, in the current analysis, no cases of stroke occurred in the ST group and three cases of stroke occurred in the DT group. In the DT group, the operator could monitor the PV signals simultaneously during ablation. Therefore, a concern regarding an increased risk of AF recurrence may exist in the ST group because of the higher possibility of missing the gap along the PV antral lesions. However, our results showed no difference in the rate of AF recurrence between the two groups. Also, our results showed no difference in the number of PV reconnection when we performed redo‐RFCA. Additionally, the rates of AF recurrence were slightly lower in the ST group than in the DT group after excluding patients using AADs. However, it is difficult to conclude that the ST approach is more effective than the DT approach in the current setting, and further studies would be needed to clarify this issue.

### 4.5 Limitations

Our study has several limitations. First, this was an observational study using a single‐center prospective registry that included a highly selected group of patients referred for AF catheter ablation. The choice of the ablation strategy depended on the operators' discretion and experience. Also, selecting the ST or DT approach was decided by operator preference and it could result in selection bias. To compensate for this limitation, we performed propensity score matching to adjust the baseline clinical characteristics of the two groups. Second, there was a significant difference in the percentage of AAD use between the two groups. Physicians tend to prescribe AADs to patients who are more likely to develop AF recurrence, such as patients with more premature atrial contractions. Therefore, this could have affected the AF‐free survival rate after the catheter ablation. Third, since our study was not randomized, the operator variable could affect the procedure and the outcome of the study. Fourth, unlike our previous report, the present study had no objective indicator to prove the reduction of discomfort because of the retrospective design.

## 5 CONCLUSION

The ST approach is as safe as the DT approach and has the advantage of reducing patient discomfort with respect to hemostasis. Although the ST approach increases the procedure time compared to the DT approach owing to the need for more frequent catheter exchanges, the ST approach is a feasible and safe strategy for AF ablation in terms of rhythm outcomes and risk of complications.

## CONFLICT OF INTEREST

The authors declare no conflicts of interest.

## Data Availability

The data of this study are available on request from the corresponding author.
